# Retraction Note: Safety and efficacy of favipiravir versus hydroxychloroquine in management of COVID-19: A randomised controlled trial

**DOI:** 10.1038/s41598-021-98683-5

**Published:** 2021-09-18

**Authors:** Hany M. Dabbous, Manal H. El‑Sayed, Gihan El Assal, Hesham Elghazaly, Fatma F. S. Ebeid, Ahmed F. Sherief, Maha Elgaafary, Ehab Fawzy, Sahar M. Hassany, Ahmed R. Riad, Mohamed A. TagelDin

**Affiliations:** 1 Tropical Medicine Department, Faculty of Medicine, Ain Shams University, Ramsis Street, Abbassia Square, Cairo, 11591 Egypt; 2 Faculty of Medicine, Ain Shams University Research Institute-Clinical Research Centre (MASRI-CRC), Cairo, Egypt; 3 Chest Department, Ain Shams University, Cairo, Egypt; 4 Community Medicine Department, Ain Shams University, Cairo, Egypt; 5 Tropical Medicine Department, Assiut University, Assiut, Egypt

Retraction of: *Scientific Reports* 10.1038/s41598-021-85227-0, published online 31 March 2021

The Editors have retracted this Article.

After concerns were brought to the Editors' attention after publication, the raw data underlying the study were requested. The authors provided several versions of their dataset. Post-publication peer review confirmed that none of these versions fully recapitulates the results presented in the cohort background comparisons, casting doubt on the reliability of the data. Additional concerns were raised about the randomisation procedure, as the equal distribution of male and female patients is unlikely unless sex is a parameter considered during randomisation. However, based on the clarification provided by the authors, sex was not considered during this process. The Editors therefore no longer have confidence in the results and conclusions presented.

Hany M. Dabbous, Manal H. El-Sayed, Gihan El Assal, Hesham Elghazaly, Fatma F. S. Ebeid, Ahmed F. Sherief, Maha Elgaafary, Ehab Fawzy, Sahar M. Hassany, and Ahmed R. Riad disagree with the retraction. Mohamed A. TagelDin did not respond to the correspondence from the Editors about the retraction.

